# Evaluation of Lead (Pb(II)) Removal Potential of Biochar in a Fixed-bed Continuous Flow Adsorption System

**DOI:** 10.5696/2156-9614-10.28.201210

**Published:** 2020-12-07

**Authors:** Pushpita Kumkum, Sandeep Kumar

**Affiliations:** Department of Civil and Environmental Engineering, Old Dominion University, Norfolk, VA, USA

**Keywords:** fixed-bed adsorption, lead, breakthrough curve, Yoon-Nelson model, biochar, Pb(II)

## Abstract

**Background.:**

Lead (Pb(II)) exposure from drinking water consumption is a serious concern due to its negative health effect on human physiology. A commercially available filter uses the adsorption potential of activated carbon for removing heavy metals like Pb(II). However, it has some constraints since it uses only surface area for the adsorption of these contaminants. Biochar produced via slow pyrolysis of biomass shows the presence of oxygen-containing functional groups on its surface that take part in the adsorption process, with higher removal potential compared to activated carbon.

**Objectives.:**

The current study examined the adsorption kinetics and mechanisms of Pb(II) removing potential of biochar from water using a fixed-bed continuous flow adsorption system.

**Methods.:**

The effect of initial Pb(II) concentration, mass of adsorbent (bed depth), and flow rate on adsorption potential were evaluated. The Adams-Bohart model, Thomas model, and Yoon-Nelson model were applied to the adsorption data.

**Results.:**

The maximum removal efficiency of Pb(II) was 88.86 mg/g. The result illustrated that the Yoon-Nelson model is the best fit to analyze the adsorption phenomena of Pb(II) in a fixed-bed biochar column.

**Conclusions.:**

The breakthrough data obtained from this study can be utilized to design a point of use filter that would be able to effectively remove Pb(II) from drinking water.

**Competing Interests.:**

The authors declare no competing financial interests.

## Introduction

Lead (Pb) is a naturally occurring heavy metal that can be found in all parts of the environment including homes. Lead and Pb-based compounds were widely used in products such as paint, ceramic, pipes, plumbing materials, solders, gasoline, batteries, ammunition, and cosmetics until the United States federal government started to discontinue its use in 1973 and banned use by 1996 because of serious health effects from Pb exposure. Lead is dangerous to all humans, but children are especially prone to experience negative impacts since their brain and nervous systems are still developing.^1^ Lead exposure can cause behavioral problems and learning disabilities, decreased intelligence quotient and hyperactivity, developmental delay, auditory problems, and anemia. Lead can be very harmful to human health even at a very low level which is why the United States Environmental Protection Agency (USEPA) does not specify a safe level of this toxic metal. The federal government banned the use of Pb-based paint in housing in 1978, but Pb-containing solder, service lines, and plumbing features are still in use. The Lead and Copper Rule regulated by the USEPA was established in 1991 and set down various requirements in order to control Pb exposure through drinking water.^2^ These regulations have some shortcomings and do not fully prevent exposure to Pb. Lead exposure through household water supply became a life-threatening issue during the Flint, Michigan water crisis. The corrosive water of the Flint river leached out lead from the pipes and contaminated the tap water supply. Some collected water samples had Pb levels more than 100 times the action level.^3^ Using an anti-corrosive agent may reduce the leaching of Pb but cannot eliminate the possibility entirely and thus can be considered a temporary solution only. Replacing all existing service lines would require billions of dollars.^4^

Studies have been conducted to evaluate the adsorption of heavy metals using different low-cost biosorbents (bacteria, microalgae, fungi, yeasts, agricultural wastes, natural residues, etc.) and polymer-based synthetic adsorbents.^5^ Chemically-treated rice husk appeared to be more efficient in removing heavy metals like Pb, arsenic (As(V)), nickel (Ni(II)), and zinc (Zn(II)) compared to untreated rice husk due to the presence of polyphenolic functional groups.^6^ Sawdust obtained from the wood industry works as a very good low-cost biosorbent and can remove Zn(II), copper (Cu(II)), chromium (Cr(VI)), and cadmium (Cd(II)).^7,8^ Khoramzadeh et al. (2013) examined mercury adsorption on sugarcane bagasse and achieved 97.584% removal efficiency.^9^ Several studies have investigated the adsorption of Cu(II) and Zn(II) by extracellular polymeric substances (EPS) extracted from sulfate-reducing bacteria and found that adsorption of Zn(II) was higher than that of Cu(II).^10^ Microalgae is capable of metal adsorption as a resistance mechanism in the presence of growth-inhibiting toxic compounds. Different species of microalgae showed high removal yield of various metals i.e., Cu(II), Ni(II), Cd(II), Zn(II) within the range of 15.4 – 836.5 mg/g.^11^ Adsorption of heavy metals over polymers such as mineral composites, phosphates composites, clay composites, metal oxide composites, were investigated by Zhao et al. (2018) and they found that these composites create strong bonds with metals and strip them off from aqueous solution.^12^

Many studies have investigated the removal of lead nitrate (Pb(II)) using biochar. Inyang *et al*. (2011) conducted a comparative study of Pb(II) adsorption between commercial activated carbon and biochar obtained from anaerobically digested sugarcane bagasse and found that biochar is more efficient in removing Pb(II) from water compared to activated carbon.^13^ Biochar has better performance compared to granular activated carbon (GAC) in the removal of Pb(II).^14^ According to a study conducted by Alhashimi in 2016,^15^ biochar production requires less energy, has less of a negative effect in climate change, and is more economical in removing metal compared to activated carbon. Surface composition (mainly abundance of oxygen-containing functional groups such as carboxyl, carbonyl, hydroxyl) predominates over the surface area in comparison with GAC in metal adsorption.^16^ Sugarcane biochar is found to be more effective compared to orange peel biochar in removing Pb(II).^17^ Biochar produced from water hyacinth biomass was utilized to investigate the adsorption capabilities of Pb(II) and Cd(II) and the maximum adsorption capacity was 123.37 mg/g for Pb(II) which is significantly higher compared to Cd(II) adsorption at 70.77 mg/g.^18^ A comparative analysis was conducted to evaluate the sorption potential of sugarcane bagasse, eucalyptus forest residues, castor meal, green coconut pericarp, and water hyacinth where sorption data reasonably fit with the Freundlich model and the cationic exchange along with specific surface complexation was found to be the dominant sorption mechanism.^19^ Xu *et al.* (2017) showed that biochar derived from waste-art-paper removed aqueous Pb(II) with extraordinary sorption capacity.^20^ The majority of these studies were conducted with a focus on batch adsorption experiments. The main purpose of running batch adsorption experiments is to evaluate the viability of the adsorbent-adsorbate system. This is more suitable for the optimization of adsorption conditions in a laboratory set-up.^21^ However, a fixed-bed column study is needed to design a flow-through adsorption system.^22–24^ Data obtained from continuous flow-through processes are able to provide guidance for scaling-up to an industrial system.

**Abbreviations**
*FTIR*: Fourier-transform infrared spectroscopy
*GAC*: Granular activated carbon

The present study was mainly focused on the Pb(II)-removing potential of biochar in a fixed bed continuous flow process to address the issue of Pb(II) contamination of drinking water. This is a novel step towards designing a point-of-use water filter exploiting the benefit of a cost-effective adsorbent like biochar. To the best of our knowledge, this is a unique approach investigating commercially available biochar for adsorbing the neurotoxic heavy metal Pb(II) in a flow-through system. Commercially available biochar can be exploited to remove Pb(II) from water in an emergency. However, biochar can be produced locally using any clean source of biomass. Household recycled items such as food cans can be used as a pyrolizer and use the produced biochar as an adsorbent for removing Pb(II). The resultant data can be utilized to design a water filter for a sustainable low-cost option for many parts of the world. The Adams-Bohart, Thomas, and Yoon-Nelson models are commonly used for predicting adsorption parameters in a column study.^25–27^ The Adams-Bohart model envisions a direct relationship between the height of the bed and the time needed for a breakthrough to be achieved. This linear connection makes the design and analysis easier and also works as a simple approach to run pilot tests.^28^ The Thomas model is a universally used theoretical model to explain the effectiveness of the column.^29^ The Yoon – Nelson model does not require any comprehensive data on adsorbate and adsorbent properties which makes it a simpler model compared to others.^30^ Because of the beneficial aspects of these models, they were applied to data collected from adsorption experiments in the present study.

## Methods

A five-gallon bag of commercially produced biochar was procured through Amazon sold by New Hampshire Biochar-The Charcola group. According to the manufacturer, the feedstock was a mix of hardwood and softwood (maple, birch, and pine). It was produced via slow pyrolysis at 500°C. The intent was to run the adsorption experiments using the biochar as-is, with no washing or alkali treatment. In order to have uniform-sized particles, the biochar was passed through 0.02–0.17 μm sieves.

### Biochar characterization

The International Biochar Initiative (IBI) developed a protocol to universally and consistently define biochar and to ensure its usability based upon the featured characteristics.^31^ “The Standardized Product Definition and Product Testing Guidelines for Biochar That Is Used in Soil” are the IBI Biochar Standards. In the present study, the characterizations of biochar follow these standards.

#### Elemental analysis

A Flash 2000 elemental analyzer was used to determine the carbon, nitrogen, and hydrogen components of the biochar sample. For quantifying the ash component, approximately 0.5 g of dried sample was weighed into ceramic crucibles and incinerated overnight in a muffle furnace at 575°C.

#### Fourier-transform infrared spectroscopy analysis

Fourier-transform infrared (FTIR) spectroscopy (4000 – 400 cm^−1^) analysis was carried out using a Bruker Alpha, Platinum-ATR instrument to identify the surface functional groups of the biochar.

#### Brunauer-Emmett-Teller surface area measurement

Brunauer-Emmett-Teller (BET) surface area analysis was conducted using the NOVA 4000e surface area and pore size analyzer (Quantachrome Instruments). Raw biochar samples were analyzed for multipoint BET surface area using nitrogen as the adsorbing gas. The analysis involved degassing the sample at 150°C for 4 h.

### Chemicals and reagents

All chemicals and reagents used for this study were of analytical grade. The Pb(II) stock solution (1000 mg/L) was prepared by dissolving 1.598 g Pb(NO_3_)_2_ (Sigma-Aldrich, Switzerland) into deionized water in 1000-mL volumetric flasks. Solutions of desired concentrations were prepared by diluting the stock solution using deionized water. The procedure for mixing the stock solution was done according to the Standard Methods for Examination of Water and Wastewater 22^nd^ edition.^32^

### Batch experiments

Two sets of batch adsorption experiments were set to evaluate the effect of the initial Pb(II) concentration. Two hundred and fifty (250)-mL Erlenmeyer flasks were used as reactors. A known amount of biochar was added to Pb(II) solutions of varying concentrations. Samples were then agitated at 180 rpm for 4 days and extracted at 0 h, 0.5 h, 1 h, 2 h, 4 h, 24 h, and 96 h using a 0.22-μm polyvinylidene fluoride filter. Collected samples were acidified to pH < 2 using (1:1) nitric acid and analyzed using an atomic absorption spectrophotometer (AA-7000) from Shimadzu. After 4 days, the pH of the solutions were measured. These experiments were performed without adjusting the pH of the mixed solution.

#### Effect of initial concentration

To evaluate the effect of initial Pb(II) concentration, 250-mL flasks of 40 mg/L, 10 mg/L, and 1 mg/L Pb(II) were chosen with a biochar dosage of 0.5 g/L in each in batch adsorption study.

#### Effect of biochar dosage

For investigating the effect of biochar dose, 10 mg/L of Pb solution at three different dosages- 0.1 g/L, 0.5 g/L, and 1 g/L of biochar were chosen for the batch adsorption studies.

### Column experiments

**Figure 1:**
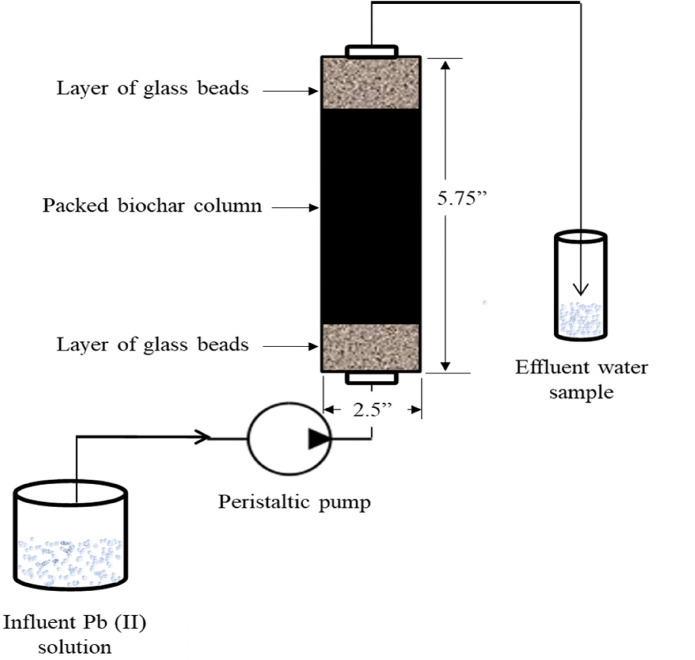
Schematic of the fixed-bed adsorption system

A fixed bed adsorption system was adopted for column-flow adsorption experiments. A schematic of the system is shown in Figure 1. Biochar was packed inside the glass column as an interlayer between two layers of glass beads. The columns were flushed with di-ionized water for 6 h before running the experiment. The Pb(II) solution prepared using the buffer solution (to keep the pH below the precipitation level) was delivered through the column by a Masterflex peristaltic pump. All the experiments were conducted in an upward flow manner to avoid canalizing in the column. Samples were collected at predetermined sampling times and acidified to pH < 2 using (1:1) nitric acid. Collected samples were analyzed using an atomic absorption spectrophotometer. Column flow experiments were conducted for an extended period of time (29 days) to reach complete saturation of the adsorption bed. Operating condition variables i.e., initial Pb(II) concentration (10 and 20 mg/L), depth of biochar column (5.72 cm, 7.62 cm), and flow rate (2 mL/min and 4 mL/min) were varied.

#### Effect of initial concentration

The stock solution of Pb(II) was diluted to 10 and 20 mg/L to evaluate the effect of initial Pb(II) concentration on the adsorption of biochar. The flow rate of the influent was adjusted at 2 mL/min and the bed depth of biochar remained at 7.62 cm (15 g biochar). Effluents were collected at certain intervals starting from 0 days to 29 days, acidified, and kept for analysis.

#### Effect of flow rate

Different flow rates can influence the adsorption of Pb(II) by biochar due to the time of exposure between Pb(II) and biochar. Influent was delivered at two different flow rates including 2 mL/min and 4 mL/min to investigate the effect of flow rate. The initial Pb(II) concentration remained constant at 10 mg/L and bed depth at 7.62 cm (15 g of biochar). Effluents were collected at certain intervals starting from 0 days to 29 days, acidified, and kept for analysis.

#### Effect of bed depth

To evaluate the effect of bed depth, varying amounts of biochar were packed inside the columns. The bed depths of the packed biochar were measured as 5.72 cm for 10 g of biochar and 7.62 cm for 15 g of biochar. Other parameters i.e., flow rate and initial Pb(II) concentration were set at 2 mL/min and 10 mg/L, respectively. Effluents were collected at certain intervals starting from 0 days to 29 days, acidified, and kept for analysis.

### Mathematical expression of batch and fixed-bed column studies

The amount of Pb(II) removed by adsorption was calculated using Equation 1:

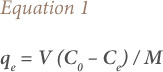
Where q_e_ is the amount of Pb(II) removed per gram of biochar, C_0_ and C_e_ are the initial and equilibrium Pb(II) concentrations (mg/L) in solution, V is the solution volume (L), and M is the dry weight of biochar in grams.

The effectiveness of the column can be evaluated using the breakthrough curve of the continuous flow system. The breakthrough curve was derived by plotting C/C_0_ vs time t, where C_0_ and C are the initial and equilibrium Pb(II) concentrations (mg/L) in solution, respectively. Total Pb(II) adsorbed by the biochar column (q_total_) can be obtained by calculating the area under the plot of the adsorbed concentration (C_ad_ = C_0_ – C (mg/L)) versus time t (min) which can be expressed by Equation 2:

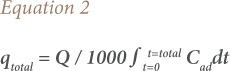
where Q is the volumetric flow rate (mL/min).

The experimental adsorption capacity q_eq(exp)_ (mg/g) can be calculated by Equation 3:

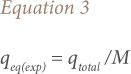
where M is the total dry weight of biochar in the column.

### Adsorption model for fixed-bed column studies

The breakthrough curves were examined by the commonly used mathematical Adams-Bohart, Thomas, and Yoon-Nelson models.

#### Adams-Bohart model

The Adams-Bohart model (24) is used for explaining the initial portion of the breakthrough curve. The equation of the Adams-Bohart model can be elaborated as:

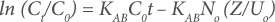
where C_0_ is the inlet adsorbate concentration, C_t_ is the outlet adsorbate concentration (both in mg/L), Z represents the bed depth (cm) of the column, U_o_ is the superficial or linear velocity (cm/min) calculated by dividing the flow rate by the column cross-section area, N_o_ is the adsorbate saturation concentration (mg/L), K_AB_ represents the kinetic constant (L/mg/min) of the model. A linear plot of ln (C/C_0_) versus time (t) was applied to determine the values of K_AB_ and N_0_.

#### Thomas model

The Thomas model (25) follows the Langmuir isotherm for equilibrium and second-order reversible reaction kinetics. The Thomas model can be expressed as:

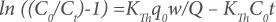



Here K_Th_ is the Thomas model rate constant (mL/mg/min), q_0_ represents equilibrium adsorbate uptake (mg/g), w is the mass of the adsorbent (g) and Q represents flow rate (mL/min). A linear plot of ln ((C_0_/C)-1) versus time (t) was applied to determine the values of K_TH_ and q_0_.

#### Yoon-Nelson model

The Yoon-Nelson model^27^ is a descriptive model that uses experimental data to estimate breakthrough parameters. The linear equation for the model can be expressed as follows:

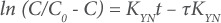
Where K_YN_ is the Yoon-Nelson rate constant (min^−1^), and *τ* represents the time required for 50% adsorbate breakthrough (min). A linear plot of ln (C/C_0_ - C) versus time (t) was applied to determine the values of K_YN_ and *τ*.

## Results

The surface characteristics of the biochar used in this study were estimated using a BET surface area analyzer. The specific surface area of the biochar was determined as 285 m^2^/g with an average pore diameter of 1.96 nm and a cumulative pore volume of 0.14 cm^3^/g which indicates that the biochar is rich in micropores.

The elemental composition of biochar used in this study is shown in Table 1.

**Table 1:** Elemental Composition of Biochar

**Component name**	**Values**
Surface area (m^2^/g)	285.40
Pore volume (cm^3^/g)	0.14
Pore diameter (nm)	1.96
PH	7.58^a^
Ash content (%)	35.48
Nitrogen	0
Carbon	44.59
Hydrogen	1.01
Sulphur	0
Oxygen	18.93^b^
H/C Molar Ratio	0.07
O/C Molar Ratio	0.16

^a^Determined following International Biochar Initiative protocol

^b^Determined by differences after retrieving data from carbon hydrogen and nitrogen (CHN) analyzer

**Figure 2:**
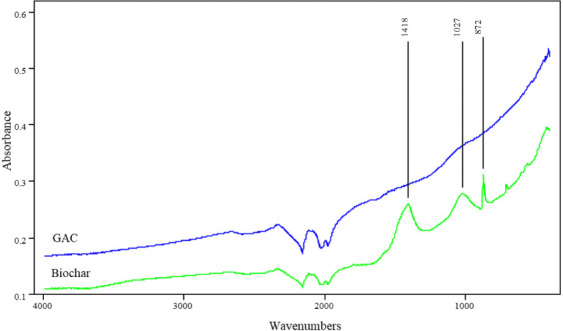
Fourier-transform infrared spectroscopy spectra of GAC and Biochar

Figure 2 shows the FTIR spectra of biochar in the near IR region (wavenumber 4000 – 400 cm^−1^). The spectra demonstrated the presence of several oxygen-containing functional groups in biochar. The peaks that can be identified and designated were aromatic C=C (~1418 cm-1), aromatic carbonyl/carboxyl C=O, phenolic –OH (~1027 cm-1), and aromatic –CH (~872 cm-1). The presence of several oxygen-containing functional groups was confirmed by Kumar *et al.*^33^ in previous research. Aromatic carbonyl/carboxyl groups exhibit high affinity with heavy metals.^16^ On the other hand, the FTIR spectra of GAC showed the absence of these functional groups.

### Batch studies

**Figure 3:**
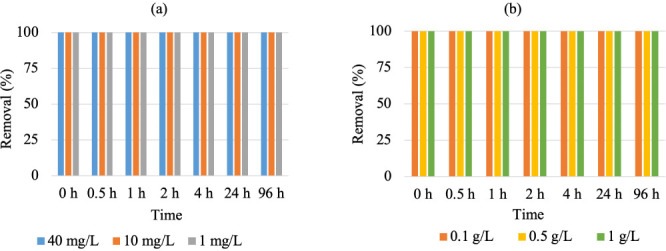
(a) Effect of initial concentration on Pb adsorption by biochar. Dosage of biochar = 0.5 g/L (b) Effect of biochar dosage on Pb adsorption. Initial Pb concentration = 10 mg/L

The batch experiments *(Figure 3a)* involving different Pb(II) concentration indicated 100% removal of initial Pb(II) concentration within the first few minutes (~ 0 h) and no trace level of Pb(II) was found even after 96 h. This demonstrates that Pb(II) adsorption to biochar was a rapid phenomenon which result from the strong inclination of Pb(II) ions to biochar leading to the fast diffusion of Pb(II) ions into the pores of the biochar to reach equilibrium.^34^ For the initial Pb(II) concentration of 40 mg/L, 10 mg/L and 1 mg/L, the equilibrium adsorption was calculated to be 80 mg/g, 20 mg/g and 2 mg/g, respectively. The experiments involving different dosage of biochar *(Figure 3b)* also showed 100% removal of Pb(II) within the first few minutes (~ 0 h). For the increased dose from 0.1 g/L to 1 g/L, the adsorption uptake decreased from 100 mg/g to 10 mg/g. This indicates that with the increase of dosage, the number of available binding sites increased but remained unsaturated since the initial Pb(II) concentration did not change. A similar phenomenon has been observed in previous studies.^3^

**Figure 4:**
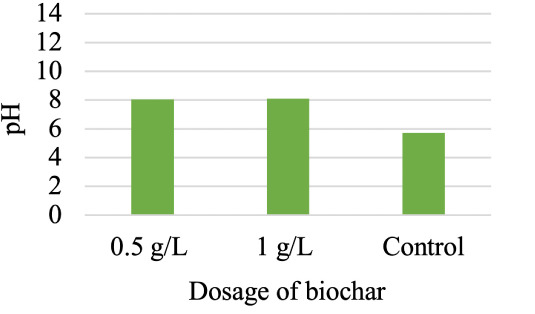
Change in pH after adding biochar in the reactorconcentration = 10 mg/L

It was also observed *(Figure 4)* that dosing with biochar increased the pH of the solution from ~ 6 to ~ 8. This could occur due to the release of inorganic alkaline species upon adding biochar to the solution. However, the high ash content of the biochar (35.48%) analyzed in this study could also be associated with the presence of these oxides and hydroxides of base cations i.e., CaO, KOH, Mg(OH)_2,_ etc.^36^ Lead ions can precipitate as hydroxides at pH ≥ 7 which led many researchers to restrict their experiments within the pH range of 2 to 6 to ensure that Pb(II) removal occurs only by adsorption.^37–39^

### Column studies

A fixed-bed column adsorption study can be used to determine the breakthrough capacity and overall life span of the adsorbent bed which can be used to design a reactor for real-time application. The provision of regulating the flow rate can be utilized for filter application and optimization of the process parameters can be useful for designing a fixed-bed adsorbent system.

The effectiveness of a fixed-bed column is evaluated by a breakthrough curve which is a demonstration of the ratio of effluent-influent concentration (C/C_0_) against time (t) profile in a continuous flow system.^40^ In the present study, the major variables for a breakthrough analysis are the bed height as well as the mass of adsorbent, flow rate, and initial inlet concentration. The soundness of the column is measured by the effects of these variables on the breakthrough curve.

#### Effect of initial Pb(II) concentration

**Figure 5—:**
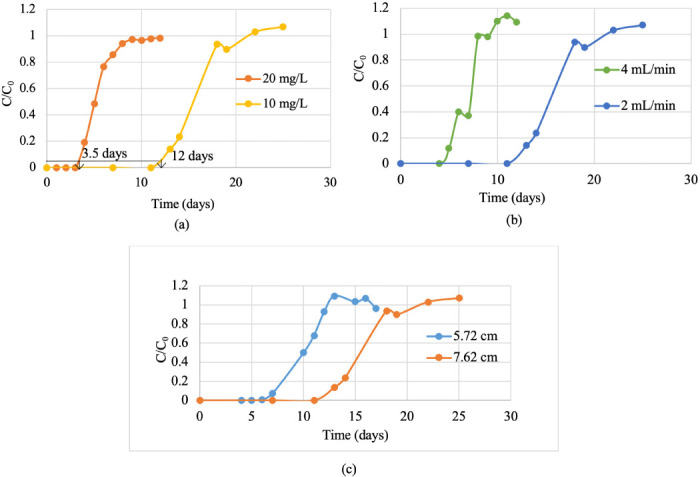
(a) Effect of initial Pb(II) concentration on breakthrough curves (bed depth= 7.62 cm, flow rate = 2 mL/min) (b) Effect of flow rate on breakthrough curves (bed depth = 7.62 cm, initial Pb(II) concentration = 10 mg/L) (c) Effect of bed depth on breakthrough curves (flow rate = 2 mL/min, initial Pb(II) concentration = 10 mg/L)

The effect of the initial Pb(II) concentration on breakthrough curves was investigated using inlet concentrations of 10 mg/L and 20 mg/L. A constant bed depth of 7.62 cm and a flow rate of 2 mL/min was maintained. The results are shown in Figure 5a and Table 2.

**Table 2:** Experimental Data of the Column Parameters Determined at Various Initial Pb(II) Concentrations

**C_0_ (mg/L)**	**Q (mL/min)**	**Mass of Adsorbent (g)**	**Z (cm)**	**pH of the effluent**	**q_total_ (mg)**	**q_eq(exp)_ (mg/g)**
10	2	10	5.72	4.29	888.55	88.86
10	2	15	5.72	4.45	1238.50	82.57
20	2	15	7.62	4.31	1274.80	84.99
10	4	15	7.62	4.50	481.78	32.12

The time to reach the breakthrough point (the point where the ratio of effluent concentration/influent concentration is equal to 0.05) decreased with increasing initial Pb(II) concentration (for 20 mg/L, the breakthrough time was approximately 3.5 days and for 10 mg/L the breakthrough time was approximately 12 days- ref. *(Figure 5a)*) which implies that the bed saturates earlier if higher amounts of Pb(II) ions were fed to the column. Decreasing breakthrough time resulted in a steeper breakthrough curve.

The mass transfer coefficient is proportionately dependent on the concentration gradient of the transporting species. Therefore, increasing the initial Pb(II) concentration would increase the concentration gradient and hence will increase the mass transfer coefficient.

Similar phenomena were observed in other studies.^22,41–43^ The equilibrium uptake of Pb(II) q_eq_ decreased and maximum adsorption q_total_ increased with increased initial Pb(II) concentration. This is an exception and not an expected trend. This finding is not in agreement with the observations of other researchers,^43,44^ but Canteli *et al.*^42^ observed a similar phenomenon in their study investigating the adsorption of benzaldehyde on activated carbon obtained from coconut husk.

#### Effect of flow rate

The effect of the flow rate on breakthrough curves was investigated using two different flow rates i.e., 2 mL/min and 4 mL/min, when a constant bed depth of 7.62 cm and an initial Pb(II) concentration of 10 mg/L was maintained. The results are shown in Figure 5b and Table 2.

As is evident from the breakthrough curve Figure 5b, increased flow rate resulted in shorter breakthrough time. Variation of the slope of the breakthrough curve for two different flow rates can be explained through mass transfer as well. Increased flow rate resulted in an increased rate of adsorbed Pb(II) onto unit bed depth which increases the mass transfer coefficient implying faster saturation. This trend was similar to other studies in the literature.^21^ A higher flow rate provides inadequate contact time between the adsorbent in the column and the Pb(II) ions which resulted in a much lower maximum and equilibrium capacity (32.18 mg/g) compared to that for decreased flow rate (88.86 mg/g). Previous studies also found similar results.^41–43^

#### Effect of bed depth

The effect of the bed depth on breakthrough curves was investigated using two different amounts of biochar 10 g and 15 g that provided varying bed depths i., e., 5.72 cm, and 7.62 cm, respectively. A constant flow rate of 2 mL/min and an initial Pb(II) concentration of 10 mg/L was maintained. The results are shown in Figure 5c and Table 2.

As seen in Figure 5c, increasing the bed depth resulted in increased breakthrough time. The slope of the breakthrough curve was slightly decreased. The broadening of the mass transfer zone may explain this.^45^ Higher bed depth implies a higher amount of biochar available for the adsorption of Pb(II). A higher amount of biochar indicates a higher surface area to be filled up with Pb(II) ions. Therefore, increasing bed depth significantly increased the breakthrough and saturation time. This observation is in line with findings by other studies.^46^

### Adsorption model for fixed-bed column studies

#### Adams-Bohart model

The Adams-Bohart model can be employed to estimate the fixed-bed parameters of maximum saturation concentration N_0_ and the Adams-Bohart rate constant K_AB_. Linear regression of all the breakthrough curves gives the value of these parameters which were calculated and presented in Table 3.

**Table 3:** Adams-Bohart Variables at Different Conditions by Linear Regression Analysis

C_0_ (mg/L)	Q (mL/min)	Z (cm)	K_AB_ (×10^6^)(L mg^−1^ min^−1^)	N_0_ (×10^−3^) (mg/L)	R^2^
20.00	2.00	7.62	5.00	3.53	0.74
10.00	2.00	7.62	20.00	1.95	0.86
10.00	4.00	7.62	20.00	2.41	0.74
10.00	2.00	5.72	20.00	3.30	0.66

With increased initial Pb(II) concentration, rate constant K_AB_ decreased and maximum adsorption capacity increased. This observation is in agreement with the results of other studies.^21^ The change in concentration gradient induces changes in rate constant and adsorption capacity which implies the diffusion process is concentration-dependent.^47^ If the flow rate increases, that implies that adsorbate was getting introduced at a higher rate, which was supposed to be inducing faster saturation, and hence K_AB_ should have decreased (minimal resistance). For this study, an increased flow rate did not show any changes, but validated the observation for increased adsorption capacity, N_0_. For varying bed depth, the value of K_AB_ did not change, but maximum adsorption capacity decreased for increased bed depth. This can be explained by the hypothesis that increased bed depth provided an increased number of available binding sites for adsorption.^44^ Overall, this model showed that higher initial Pb(II) concentration, flow rate, and bed depth will increase the adsorption potential of biochar. The R^2^ values for the Adams-Bohart model are low, ranging from 0.66 to 0.86, which indicates that this model is not a good fit for this study.

#### Thomas model

The simplified expression of the Thomas model was used to predict the unknown values of the Thomas rate constant. The variables K_TH_ and q_0_ and the linear regression coefficient (R^2^) are presented in Table 4.

**Table 4:** Thomas Variables at Different Conditions by Linear Regression Analysis

C_0_ (mg/L)	Q (mL/min)	Z (cm)	K_TH_ (×10^3^)(mL mg^−1^ min^−1^)	q_0_ (mg/g) (calculated)	q_0_ (mg/g) (experimental)	R^2^
20.00	2.00	7.62	20.00	28.47	49.92	0.93
10.00	2.00	7.62	50.00	31.08	24.96	0.95
10.00	4.00	7.62	110.00	25.20	49.92	0.84
10.00	2.00	5.72	50.00	56.59	37.44	0.96

From the calculated values for Thomas model parameters, it can be said that rate constant and equilibrium adsorbate uptake both decreased with the increase of initial Pb(II) concentration, which implies that the concentration gradient was the impelling cause for adsorption. This is similar to studies conducted by other researchers.^44,48,49^ With increased flow rate, the rate constant increased which indicates there was a lack of time for the contaminants to transfer from the liquid phase to the solid surface of biochar, and hence adsorbate uptake decreased.^50^ Increased bed depth did not have much impact on rate constant, but adsorbate uptake decreased which indicates there were more binding sites available for adsorption. Table 4 provided the comparative values for calculated q_0_ using the model and the experimental q_0_. According to the Thomas model, lower initial Pb(II) concentration, flow rate, and bed depth will increase the adsorption of Pb(II) on biochar. As seen in the table, there was a 20–50% discrepancy between the predicted and experimental values. Even though this model demonstrated a positive correlation compared to the Adams-Bohart model (R^2^ values ranging from 0.84 to 0.96), for this study, the discrepancy between the predicted and calculated value did not support this model being a good fit. According to López-Cervantes *et al*. the main limitation of this model is that it was derived based on the assumption that biosorption follows second-order kinetics and does not depend on the transformation of reactant-product but is dominated by the mass transfer.^21^ This assumption might not be valid for processes under all varying conditions.

#### Yoon-Nelson model

The linearized form of the Yoon-Nelson model was used to estimate the values of rate constant K_YN_ and 50% breakthrough time, . The parameters value and linear regression coefficient are presented in Table 5.

**Table 5:** Yoon-Nelson Variables at Different Conditions by Linear Regression Analysis

C_0_ (mg/L)	Q (mL/min)	Z (cm)	K_YN_ (×10^2^)(min^−1^)	τ (min) (calculated)	τ (min) (experimental)	R^2^
20.00	2.00	7.62	0.03	12104.30	10080.00	0.93
10.00	2.00	7.62	0.05	23314.00	23040.00	0.95
10.00	4.00	7.62	0.11	9449.09	10800.00	0.84
10.00	2.00	5.72	0.05	28296.00	14400.00	0.96

Table 5 provides the 50% breakthrough time achieved by the prediction from the model and the actual experimental 50% breakthrough time is also shown. Increased initial Pb(II) concentration decreased the rate constant and 50% breakthrough time which implies higher feed concentration accelerated the adsorption process that provided a shorter breakthrough time. Increased flow rate gave an increased rate constant and decreased breakthrough time. Increased bed depth shortens the breakthrough time which contradicts experimental data. A comparative analysis can be made between the predicted 50% breakthrough time and experimental 50% breakthrough time which showed that the values were close to each other with no significant difference except for the condition where 10 mg/L of initial Pb(II) concentration, 2 mL/min of flow rate and 10 g of the mass of adsorbent was used. From the predicted and experimental values of the breakthrough time and listed correlation coefficient (R^2^) that ranged from 0.84 to 0.96, it can be said that the Yoon-Nelson model demonstrated the most suitable relation to the experimental data and can be utilized to portray the adsorption potential of Pb(II) in a fixed-bed biochar column.

## Discussion

Pyrolysis temperature plays an important role in the yield of biochar as well as pore size and volume of the produced biochar.^51,52^ Basic components within the biomass such as lignin, cellulose, hemicellulose break down at different stages of pyrolysis. At a temperature range of 200 – 260*°*C, hemicellulose starts to decompose, cellulose starts breaking down at 240 – 350*°*C and lignin starts to degrade from 280 – 500*°*C.^51^ It was found that at a lower pyrolysis temperature (300 – 450*°*C), the specific surface area of produced biochar is typically low (4 – 23 m^2^/g)^54^ which could be due to the blockage of the residual pores by inorganic material present in biomass.^55^ A substantial increase of surface area begins at 400 – 500*°*C^56^ (25 – 300 m^2^/g) which could be due to the degradation of organic matter and the emergence of porous or narrow passage-like structures.^57,58^ The biochar used in this study was produced at 500*°*C, in accordance with the investigation of temperature needed for yielding high surface area. Biochar produced at a temperature of over 400*°*C appeared to be more able to adsorb organic and inorganic contaminants owing to its high surface area and appearance of micropores while preserving the oxygen functional groups during pyrolysis.^59^ The higher surface area, which is also related to pore volume, influences the capacity of cation adsorption of biochar onto its surface.^60–63^ Previous studies conducted by various researchers reported the physical properties of biochar and experimental data of the adsorption experiment. Alhashimi and Aktas^15^ conducted a sensitivity analysis focusing on the effect of particle size, surface area, pH, contaminant concentration, adsorbent dose, and contact dose on the adsorption capacity of such studies. They found surface area to have the most reasonable degree of correlation with adsorption capacity compared to other properties. A similar occurrence was noted in the present study as well. The pH of slow pyrolysis biochar is typically in the range of 4 to 10.^64^ Yuan J-H *et al.* observed that biochar pH increases (from 6.5 to 10.8) with increasing pyrolysis temperature (from 300 to 700*°*C), perhaps owing to the increased accumulation of undecomposed inorganic compounds and the formation of basic surface oxides.^61,65^ According to Ronsse *et al.*, Spokas *et al.* and Zhao *et al.*, this increase in pH with increased temperature is correlated with the presence of higher amounts of ash and oxygen-containing functional groups.^54,58,64^ The value of pH (7.58) and ash content (35.48%) of the biochar used in the present study (pyrolysis temperature 500*°*C) are in accordance with their proposition. Rafiq *et al.* found increased ash content (from 5.7 to 18.7%) with an increase of temperature (from 300 – 500*°*C), which is due to the continuous congregation of minerals and absolute alteration of ligno-cellulosic components.^50,66,67^ However, the presence of foreign materials could also be responsible for high ash content, which was also observed by Lee *et al.*^68^ The elemental composition of biochar varies widely depending upon the type of feedstock and production process parameters. Slow pyrolysis biochar derived from various feedstock showed carbon content roughly in the range of 6 – 90%, oxygen content ranging from 1 – 37.7%, hydrogen content ranging from 1 – 9.9%, and nitrogen content ranging from 0.1 – 7.4%.^64^ Some studies found an increase of carbon content (from 62.2 to 92.4%) with an increase of pyrolysis temperature, which can be attributed to the extended level of chemical change eventually forming robust carbon composition.^69–71^ On the other hand, another study by Cantrell *et al*. found a decrease in carbon, hydrogen, and oxygen content ranging from 59.94 to 37.94%, 2.91 to 0.99% and from 2.13 to 0.99%, respectively, with an increase of temperature from 300 to 500*°*C and attributed this loss to the disappearance of hydroxyl and carboxyl groups of cellulose, hemicellulose, and lignin.^72,73^ The H/C molar ratio (0.07) describes the aromaticity and permanence of the biochar, whereas the O/C molar ratio (0.16) explains the hydrophilicity or abundance of polar oxygen-containing surface functional groups of the biochar, which actively take part in adsorption of heavy metals.^73^ When comparing two different biochar, lower H/C and O/C ratio indicates higher aromaticity and lower affinity to water.^74^

The intention in the present study was to keep the pH unaltered so experiments were conducted accordingly. Increased pH (up to pH = 8) may have some impact on the Pb(II) removal capacity of biochar. High pH value may facilitate removal through precipitation along with adsorption.^75^ Jalali and Aboulghazi showed that at pH ≥ 6.01, more hydroxide complexes i.e., Pb(OH)^+^, Pb(OH)_2_ easily formed and reduced the concentration of free Pb ions in the solution.^76^ Moreover, when solution pH increases, the concentration of H^+^ decreases. To balance the concentration in solution, the oxygen functional groups on the biochar surface release the negative charge and increase the electrostatic attraction for Pb(II).^77^ Comprehensive characterization of biochar before and after the experiment could provide a thorough understanding of the adsorption mechanism of Pb(II). According to Li *et al.* net release of cations i. e., Na^+^, K^+^, Ca^2+^ and, Mg^2+^ can be measured to estimate the extent of cation exchange during Pb(II) adsorption.^78^ Complexation with the oxygen functional groups on the biochar surface can be evaluated by investigating the peak area, peak intensity, and position of the peak in FTIR after Pb(II) adsorption. As stated earlier, precipitation is one of the most important mechanisms in Pb(II) removal and this can be evaluated by X-ray diffraction scanning of Pb(II) loaded biochar.

Batch experiments in this study did not lead to a point where the isotherm model could be plotted or reaction kinetics could be determined since Pb(II) was completely removed by biochar within the very first moment (~ 0 h) of the experiment. A fixed-bed column study was the main focus of this work, hence no further batch adsorption experiments were conducted.

To scale-up the system using the laboratory experimental data, two parameters have significant importance: the superficial velocity and contact time (empty-bed contact time). Both of these parameters need to be the same so that the mass transfer characteristics do not change. Superficial velocity is equal to the flow rate divided by the cross-sectional area. Therefore, using the superficial velocity and flow rate of the lab-scale system, a new value of the cross-sectional area of the packed column can be calculated which will further provide the required diameter of the column. The empty-bed contact time is equal to the volume of the bed occupied by the adsorbent divided by the flow rate and this is a function of both superficial velocity and bed depth. The required mass of the adsorbent can be calculated by multiplying the volume of the packed bed and density. This makes the density of the adsorbent an important variable, because if the particle size changes, density will also be changed which in turn will change the length of the mass transfer zone. If the mass transfer zone is short, the adsorbent bed will be fully utilized at breakthrough. This is called the point of exhaustion when the bed completely loses the ability to remove adsorbate. The rate of exhaustion can be expressed as the amount of adsorbent used in terms of a unit volume of water treated until the breakthrough. If superficial velocity increases, the length of the mass-transfer zone increases, and the rate of exhaustion decreases. Specific throughput is the volume of water treated per unit mass of adsorbent used. This is a useful variable to determine the adsorption capacity of a fixed-bed system. Thus, the column diameter of the scaled-up system can be calculated from the required throughput and the chosen velocity.

## Conclusions

Removal of Pb(II) using biochar is conducted in a fixed-bed column system by varying the initial concentration, flow rate, and bed depth to evaluate the potential of biochar in removing Pb(II) from drinking water. Different parameters effect the breakthrough and saturation capacity for Pb(II) on biochar. Increased initial Pb(II) concentration, flow rate, and decreased bed depth accelerate the process of exhaustion of the column. The column adsorption kinetic models are implemented to the experimental data to further analyze the adsorption mechanism. The Adams-Bohart model does not fit well with the experimental data with respect to both the predicted values and correlation coefficient. The Thomas model shows a good correlation coefficient but the predicted values for equilibrium adsorbate uptake have a 20–50% discrepancy with the experimental data. The Yoon-Nelson model assesses the 50% breakthrough for the column that is close to the values found through the experiment and this model also showed a good correlation between the values.
